# Weekly Intramuscular Injection of Levothyroxine following Myxoedema: A Practical Solution to an Old Crisis

**DOI:** 10.1155/2015/169194

**Published:** 2015-11-05

**Authors:** Peter N. Taylor, Arshiya Tabasum, Gina Sanki, David Burberry, Brian P. Tennant, James White, Onyebuchi Okosieme, Andrew Aldridge, Gautam Das

**Affiliations:** ^1^Endocrinology and Diabetes Department, Prince Charles Hospital, Cwm Taf University Health Board, Merthyr Tydfil CF47 9DT, UK; ^2^Thyroid Research Group, Institute of Molecular and Experimental Medicine, Cardiff University School of Medicine, Cardiff CF144XN, UK; ^3^Biochemistry Department, Prince Charles Hospital, Cwm Taf University Health Board, Merthyr Tydfil CF47 9DT, UK; ^4^Department of Medicine of the Elderly, Prince Charles Hospital, Cwm Taf University Health Board, Merthyr Tydfil CF47 9DT, UK; ^5^Pharmacy, Prince Charles Hospital, Cwm Taf University Health Board, Merthyr Tydfil CF47 9DT, UK

## Abstract

An 82-year-old female with known hypothyroidism was admitted to hospital after being found on the floor. On examination, she was unkempt, confused, bradycardic, hypothermic, and barely arousable. Initial biochemistry revealed a thyroid stimulating hormone (TSH) of >100 mU/L and free thyroxine (FT_4_) level of 1.5 pmol/L which supported a diagnosis of myxoedema coma. She was resuscitated and commenced on liothyronine, levothyroxine, and hydrocortisone and some improvement was made. It became apparent that she was hiding and spitting out her oral levothyroxine including levothyroxine elixir. Given the need for prompt alternative control, we sought advice from international experts where intramuscular levothyroxine was recommended. She was managed from day 50 onwards with intramuscular levothyroxine 200 mcg once a week, which was subsequently increased to 500 mcg. Thyroid function normalized and she made continual cognitive and physical progress and was discharged to a rehabilitation hospital. Her intramuscular levothyroxine was stopped and she was subsequently restarted on oral levothyroxine, with a plan for on-going close monitoring of her thyroid function. This report highlights the potential to use intramuscular levothyroxine in individuals with severe hypothyroidism arising from poor compliance with levothyroxine treatment or other potential causes such as impaired absorption.

## 1. Introduction

Myxoedema coma is a rare endocrine emergency with a high mortality rate of 25–70% [1]. It is uncommon nowadays due to widespread thyroid function testing and a low threshold for initiating levothyroxine therapy [2]. It has been reported that even after 5 years of levothyroxine therapy 21.5% of individuals persist in having a TSH level >5.0 mU/L [2] indicating widespread poor compliance or malabsorption. Myxoedema coma is more common in the winter months and in patients with poor compliance who are already known to have hypothyroidism [1]. Judicious use of levothyroxine, liothyronine, and hydrocortisone combined with supportive care is the mainstay of treatment for a favourable outcome.

In this paper we present an interesting case of myxoedema coma which was successfully managed with intramuscular levothyroxine as the compliance of the oral levothyroxine was poor. This is the first report which demonstrates the role of intramuscular levothyroxine in the management of myxoedema coma.

## 2. Case Report

An 82-year-old female was admitted in winter after being found unresponsive. She had a history of hypothyroidism and was prescribed levothyroxine 75 *μ*g daily. Her sister reported that over the last few months she had been increasingly tired and forgetful and was not compliant with her levothyroxine tablets.

On examination, her facies were consistent with myxedema and she was also found to be unkempt with evidence of self-neglect. She was bradycardic with a heart rate of 40 beats per minute and was profoundly hypothermic with a recorded temperature of 32.3 degrees Celsius and was barely arousable. Initial laboratory tests revealed a thyroid stimulating hormone (TSH) of >100 mU/L (0.27–4.2 mU/L) and free thyroxine (FT_4_) of 1.5 pmol/L (11–25 pmol/L). She was resuscitated with warm fluids, oxygen, and a bear hugger and was given intravenous liothyronine and oral levothyroxine (initially via nasogastric tube) along with intravenous hydrocortisone in suspicion of a possibility of coexisting adrenal suppression.

**Figure 1:**
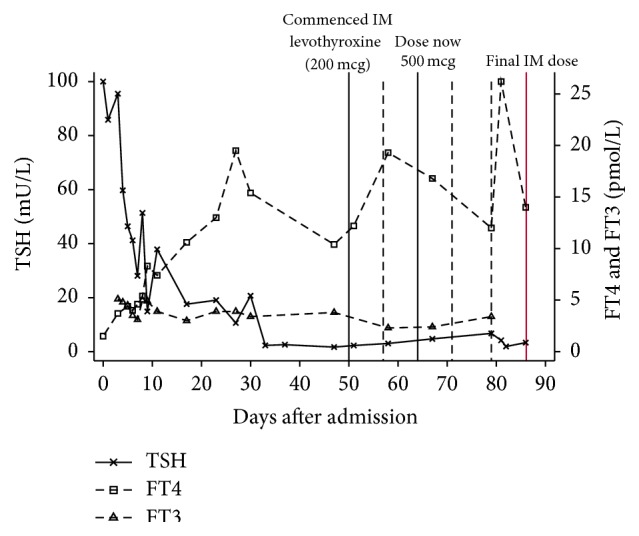
Thyroid function tests following admission. Intramuscular levothyroxine continued from day 50 until day 86 (end of graph) when converted to oral levothyroxine. Unbroken vertical lines refer to intramuscular levothyroxine initiation, dose change, and final IM dose. Dashed vertical lines refer to dates of other intramuscular levothyroxine injections.

Her clinical condition gradually improved over the next few days. Hypothermia resolved within 24 hours and her heart rate improved over a period of 48 hours. In due course her TSH began to normalize but her FT_4_ levels remained inconsistent (Figure 1). She was therefore maintained on intravenous liothyronine for a longer period than that originally planned (up to day 38) as there were concerns regarding her compliance with oral tablets even within the hospital premises. It was highlighted by the ward nurses that she was regularly hiding her tablets underneath her pillow or spitting them out even if intake was tried with supervision. This was attributed to her poor cognitive state and ongoing impaired intellectual functioning. A further attempt was made with levothyroxine elixir, but unfortunately it was unsuccessful as well.

After significant deliberation and failed attempts to stabilize her TFTs on oral medications we embarked on trying her on a weekly intramuscular levothyroxine (200 mcg) regime. This resulted in more stable thyroid function (Figure 1). She did not report any side effects from intramuscular levothyroxine or symptoms of thyrotoxicosis. Her thyroid function stabilized over the next few weeks and she continued to make significant improvement with her physical and cognitive functions. Buoyed by her dramatic improvement we increased her dose to 500 mcg once a week on the 64th day and her TFTs were repeated on a weekly basis to monitor the change. One week later she was discharged to a community rehabilitation hospital, where she continued to make good progress. After almost 3 months of levothyroxine therapy, there was a modest improvement in her cognitive state to an extent which would sufficiently enable her to take her oral medication more reliably. As her TFTs and clinical state were progressing satisfactorily we stopped her intramuscular levothyroxine on day 86 and oral levothyroxine was recommenced. Our long term plan is to manage her with supervised once weekly oral dosing or consider her for once/twice weekly intramuscular levothyroxine therapy if her thyroid function becomes suboptimal again.

Poor compliance with medications which ought to be taken lifelong is a well-recognised problem [3]. Inadequate intake of levothyroxine which is usually designed for daily intake is reflected on TFTs with a persistently elevated TSH. One strategy in treating such cases hypothyroidism has been the use of once or twice weekly supervised oral therapy, although successful studies to date have been small [4, 5]. Previous case reports have successfully demonstrated the use of intramuscular levothyroxine therapy in patients with uncharacterized malabsorption [6], but use of parenteral levothyroxine therapy has not been studied rigorously to date and there are no well-established consensus guidelines at present [6].

Our strategy of a weekly intramuscular levothyroxine therapy did appear to stabilize our patient's thyroid status and allowed her clinical and cognitive state to improve continuously. It also provided us with a window of opportunity to ensure compliance when oral preparations were proving to be difficult. Moreover, whilst there was substantial variability in her thyroid function, we could reliably stabilize her TFTs without making her overtly hyperthyroid and further detrimental effects of ongoing hypothyroidism were suitably averted. To our knowledge this is the first time that the intramuscular route has been used to deliver levothyroxine in a patient with myxoedema coma. In individuals with such profound thyroid disturbance, poor circulation with impaired transport of intramuscular levothyroxine into the circulation might have been expected, but this did not appear to be the case in our patient.

In summary this case report crucially highlights that the intramuscular route for levothyroxine is a viable strategy even in profound hypothyroidism and would be useful in patients with either poor compliance or absorption. Although there was substantial variability in FT_4_ levels on IM levothyroxine this may have been reduced by using twice weekly regime which we would consider if she requires IM levothyroxine again or when we encounter a similar patient.

## 3. Learning Point for Clinicians

This case report highlights the potential for severe harm that may arise due to poor compliance with thyroid hormone replacement. It also demonstrates that intramuscular levothyroxine is a viable treatment even in severe hypothyroidism and maybe particularly useful in individuals with poor compliance due to self-neglect and cognitive impairment or in patients with poor gastrointestinal absorption.
